# *Euphorbia tirucalli* Latex Ingestion Modifies Heart Function and Increases Myocyte Levels of Oxidative Stress in Normotensive Rats

**DOI:** 10.3390/ijms27093730

**Published:** 2026-04-22

**Authors:** Maria Eduarda De Souza Barroso, Edgar Hell Kampke, Rafaela Aires, Silas Nascimento Ronchi, Antonio Ferreira de Melo, Luciana Polaco Covre, Bianca Prandi Campagnaro, Ricardo Machado Kuster, Silvana Santos Meyrelles

**Affiliations:** 1 Graduate Program in Physiological Sciences, Federal University of Espirito Santo, Vitoria 29047-105, ES, Brazil; 2 Graduate Program in Pharmaceutical Sciences, Vila Velha University (UVV), Vila Velha 29102-920, ES, Brazil; 3 Graduate Program in Health Sciences, University Center of Espirito Santo (UNESC), Colatina 29703-900, ES, Brazil; 4 iNOVA4HEALTH, NOVA Medical School, School of Medical Sciences (NMS/FCM), NOVA University of Lisboa, 1159-056 Lisboa, Portugal; 5 Division of Medicine, University College London, London WC1E 6BT, UK; 6 Infectious Diseases Center, Federal University of Espirito Santo, Vitoria 29047-100, ES, Brazil; 7 Department of Chemistry, Federal University of Espirito Santo, Vitoria 29047-105, ES, Brazil

**Keywords:** *Euphorbia tirucalli*, cardiac hemodynamics, oxidative stress, inflammatory activity, apoptosis

## Abstract

*Euphorbia tirucalli*, commonly known as Aveloz, is widely used in Brazilian folk medicine for its purported antibacterial, antiviral, and antitumoral properties. However, scientific evidence regarding its systemic in vivo effects, particularly on the cardiovascular system, remains limited. This study investigated the impact of oral *E. tirucalli* latex ingestion on cardiac hemodynamics and associated molecular alterations in normotensive Wistar rats. Animals received water (control) or *E. tirucalli* latex (13.47 mg/kg) by oral gavage for 15 days. Hemodynamic parameters were assessed through noninvasive blood pressure monitoring and direct measurements of left ventricular systolic (LVSP) and end-diastolic pressures (LVEDP), cardiac cycle duration, rates of pressure development (dP/dT_max_ and dP/dT_min_), and the left ventricular relaxation constant (Tau). Oxidative stress and inflammation were evaluated by plasma advanced oxidation protein products (AOPP) and myeloperoxidase (MPO), respectively, while reactive oxygen species production and apoptosis were analyzed in isolated cardiomyocytes. Although systemic blood pressure remained unchanged, *E. tirucalli* increased LVSP, LVEDP, cardiac cycle duration, and dP/dT_max_, while reducing Tau. These alterations were accompanied by elevated AOPP and MPO levels, increased cardiomyocyte hydrogen peroxide, and higher rates of early apoptosis, indicating that *E. tirucalli* latex alters cardiac hemodynamics and promotes oxidative and inflammatory cardiac injury.

## 1. Introduction

The use of natural products, particularly medicinal plants, remains widespread across the globe. Every day, thousands of individuals incorporate these substances into their routines in search of therapeutic benefits. Conditions such as gastrointestinal disturbances, insomnia, anxiety, and cardiovascular diseases are frequently managed using herbal remedies [1,2,3]. The persistence of this practice across generations is largely rooted in traditional knowledge, which often endures despite the lack of rigorous scientific validation concerning efficacy and safety [4].

Among the plants that have recently gained scientific attention for their diverse bioactivities is *Euphorbia tirucalli* Linn., commonly known as Aveloz. A member of the Euphorbiaceae family, *E. tirucalli* is native to Africa but has achieved broad geographical distribution, including extensive cultivation in Brazil, where it is also used as an ornamental plant [5]. The plant’s latex has been traditionally employed as a laxative and for its purported antimicrobial and antiparasitic properties. In folk medicine, it is widely used for the treatment of warts, asthma, syphilis, and even cancer [6,7].

Driven by its popular use, numerous studies have investigated the pharmacological properties of *E. tirucalli* and its secondary metabolites. Findings suggest the plant exhibits antinociceptive and pro-angiogenic activities and may exert antitumoral effects, attenuate cachexia, and modulate immune responses [8,9,10,11]. Nevertheless, concerns have been raised regarding its safety. The latex of *E. tirucalli* shows caustic properties that can cause severe dermatological and ocular injuries, including dermatitis, conjunctivitis, and even blindness upon ocular exposure [12]. Moreover, significant pro-inflammatory responses, such as increased cytokine expression, have also been reported [13].

These findings highlight the paradox associated with medicinal plants: while widely used for their therapeutic potential, they are not exempt from eliciting adverse effects through interactions with multiple physiological systems, ultimately compromising systemic homeostasis [14,15].

Despite the promising biological effects attributed to *E. tirucalli*, most existing studies are limited to in vitro approaches and do not adequately address its systemic impact. Given the increasing popular use of Aveloz for a wide range of conditions, including cancer, and the current gaps in understanding its interactions with key physiological systems, this study aims to evaluate the effects of oral ingestion of *E. tirucalli* latex on cardiac hemodynamics, thereby mimicking its traditional use.

## 2. Results

### 2.1. Chemical Profiling of Euphorbia tirucalli Latex by GC–MS and FT-ICR MS

**Figure 1:**
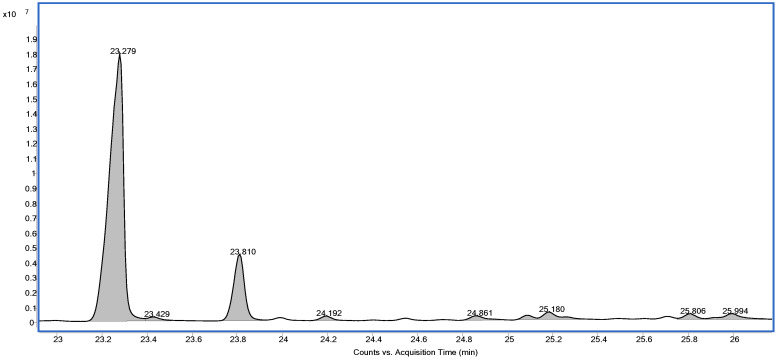
GC–MS chromatogram showing the triterpenes detected in *E. tirucalli* latex.

The chemical analysis of *Euphorbia tirucalli* latex revealed the presence of two major triterpenes. The mass spectra of both compounds, euphol (retention time: 23.279 min) and tirucallol (retention time: 23.810 min), were identical, as these molecules are stereoisomeric structures (Figure 1). The remaining minor peaks observed in the chromatogram correspond to less abundant triterpenes present in the latex.

Subsequently, under ultra-high-resolution mass spectrometry conditions using electrospray ionization in negative mode (ESI(–) FT-ICR MS), two major diterpenes were detected: ingenol and 4-deoxyphorbol. The diterpenic alcohols ingenol, phorbol, and 4-deoxyphorbol have previously been described in *E. tirucalli* and, through natural esterification processes, give rise to a variety of diterpene esters (Table 1).

### 2.2. Effects of E. tirucalli (Aveloz) Treatment on Blood Pressure and Cardiac Hemodynamics

**Figure 2:**
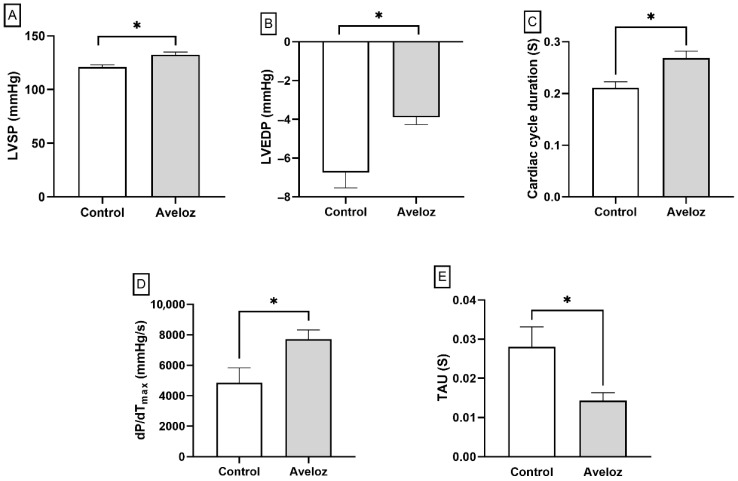
Graphs show left ventricular systolic pressure (LVSP) (**A**), left ventricular end-diastolic pressure (LVEDP) (**B**), cardiac cycle duration (**C**), maximal rate of pressure development (dP/dT_max_) (**D**), and left ventricular relaxation constant (Tau) (**E**) in control rats (white bars, *n* = 8) and *E. tirucalli*-treated rats (gray bars, *n* = 8). Data are expressed as mean ± SEM. * *p* < 0.05 versus the control group.

Figure 2A–E show that the Aveloz latex treatment significantly increased LVSP (121.1 ± 1.92 mmHg vs. 132.4 ± 2.54 mmHg) and LVEDP (−6.74 ± 0.80 mmHg vs. −3.89 ± 0.38 mmHg) values in the *E. tirucalli*-treated group compared to the control group. Interestingly, a significant increase in cycle duration (0.21 ± 0.01 s vs. 0.27 ± 0.01 s) and dP/dT_max_ (4849 ± 983.2 mmHg/s vs. 7707 ± 612.0 mmHg/s) was also observed in the *E. tirucalli* group compared to control. On the other hand, a significant reduction in the Tau constant (0.028 ± 0.005 s vs. 0.014 ± 0.002 s) was found in the *E. tirucalli* group relative to control.

Noninvasive tail-cuff measurements at the end of *E. tirucalli* treatment did not show significant differences in systolic blood pressure between control and *E. tirucalli*-treated rats (BP 117.8 ± 7.47 mmHg vs. 122.2 ± 10.34 mmHg). However, invasive hemodynamic analysis demonstrated marked alterations in left ventricular performance following *E. tirucalli* administration. As shown in Figure 2, *E. tirucalli*-treated rats exhibited significantly higher left ventricular systolic pressure (LVSP; 132.4 ± 2.54 mmHg vs. 121.1 ± 1.92 mmHg; *p* < 0.05) and elevated left ventricular end-diastolic pressure (LVEDP; −3.89 ± 0.38 mmHg vs. −6.74 ± 0.80 mmHg; *p* < 0.05) compared with controls.

Additionally, *E. tirucalli* increased the cardiac cycle duration (0.27 ± 0.01 s vs. 0.21 ± 0.01 s; *p* < 0.05) and the maximal rate of pressure development (dP/dT_max_; 7707 ± 612.0 mmHg/s vs. 4849 ± 983.2 mmHg/s; *p* < 0.05). Conversely, the left ventricular relaxation constant (Tau) was significantly reduced in the treated group (0.014 ± 0.002 s vs. 0.028 ± 0.005 s; *p* < 0.05). Collectively, these findings indicate that oral *E. tirucalli* latex ingestion modifies ventricular pressure dynamics and diastolic function without altering systemic arterial pressure.

### 2.3. Plasma Oxidative Stress and Inflammatory Markers

**Figure 3:**
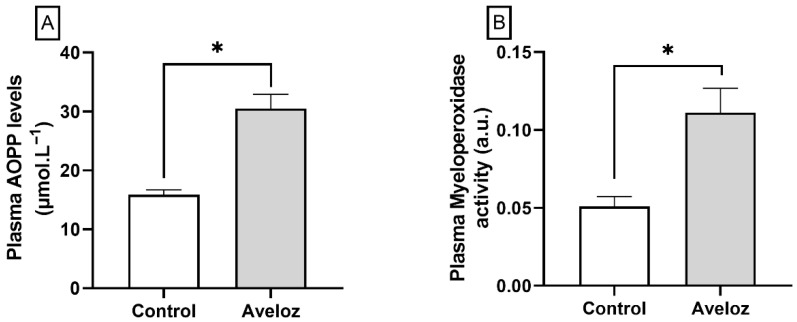
(**A**) Plasma levels of advanced oxidation protein products (AOPP) as an index of systemic oxidative stress, and (**B**) plasma myeloperoxidase (MPO) activity as an index of inflammatory response in control rats (white bars, *n* = 8) and *E. tirucalli*-treated rats (gray bars, *n* = 8). Data are expressed as mean ± SEM. * *p* < 0.05 versus the control group.

Analysis of plasma oxidative and inflammatory biomarkers revealed a significant increase in advanced oxidation protein products (AOPP) following *E. tirucalli* administration, indicating enhanced systemic protein oxidation (30.48 ± 2.42 µmol.L^−1^ vs. 15.86 ± 0.82 µmol.L^−1^; *p* < 0.05; Figure 3A). In parallel, myeloperoxidase (MPO) activity, a marker of inflammatory response, was markedly elevated in the *E. tirucalli* group compared with controls (0.111 ± 0.0156 a.u. vs. 0.051 ± 0.0006 a.u.; *p* < 0.05; Figure 3B). These results demonstrate that oral ingestion of *E. tirucalli* latex induces a pro-oxidant and pro-inflammatory systemic profile.

### 2.4. Reactive Oxygen Species (ROS) Generation and Apoptosis in Circulating Leukocytes and Cardiac Cells

**Figure 4:**
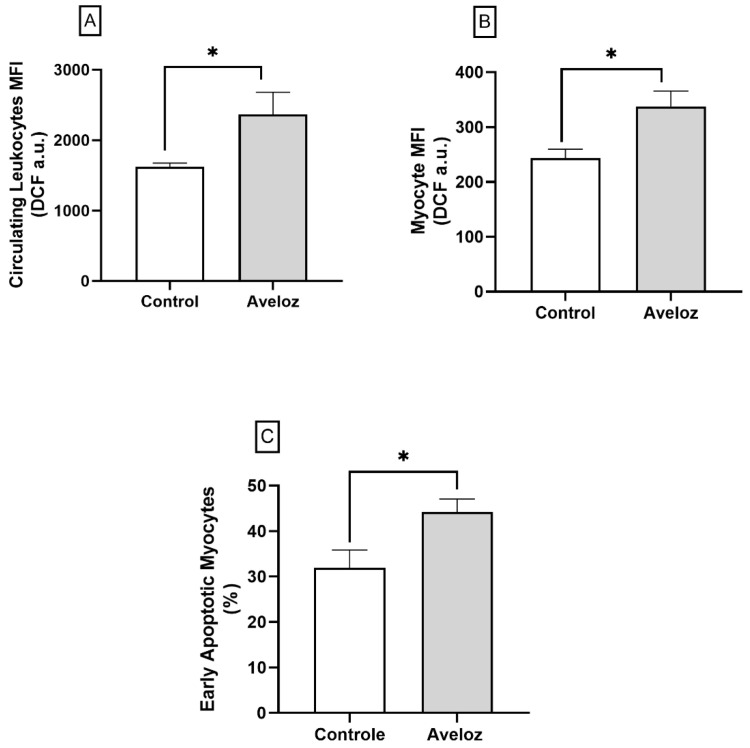
(**A**) Intracellular ROS production in circulating leukocytes, (**B**) ROS production in isolated cardiomyocytes, and (**C**) percentage of early apoptotic cardiomyocytes determined by Annexin V–FITC/propidium iodide staining in control (white bars, *n* = 8) and *E. tirucalli*-treated rats (gray bars, *n* = 8). Data are expressed as mean ± SEM from median fluorescence intensity (MFI, arbitrary units) or percentage of total cells. * *p* < 0.05 versus the control group.

Flow cytometric analysis revealed that *E. tirucalli* significantly increased intracellular reactive oxygen species (ROS) production in both circulating leukocytes and cardiac cells. As shown in Figure 4A, circulating leukocytes from treated rats exhibited higher hydrogen peroxide-dependent fluorescence intensity compared with controls (2366 ± 314.4 MFI a.u. vs. 1624 ± 52.8 MFI a.u.; *p* < 0.05). Similarly, cardiomyocytes isolated from treated animals showed increased ROS generation (337.5 ± 28.0 MFI a.u. vs. 243.6 ± 16.0 MFI a.u.; *p* < 0.05; Figure 4B).

Consistent with enhanced oxidative stress, *E. tirucalli*-treated rats presented a higher proportion of early apoptotic cardiomyocytes, as determined by Annexin V-positive/PI-negative staining (44.17 ± 2.88% vs. 31.97 ± 3.89%; *p* < 0.05; Figure 4C). These data indicate that *E. tirucalli* exposure promotes ROS overproduction and apoptosis in cardiac tissue, suggesting oxidative stress as a major driver of myocardial injury.

## 3. Discussion

Although popular culture has long recognized the therapeutic properties of *Euphorbia tirucalli*, the plant has recently garnered increased attention due to its potential antitumor activity. However, the indiscriminate use of *E. tirucalli* raises concerns regarding its actual therapeutic potential and possible interactions with physiological systems. Considering that certain plant-derived bioactive metabolites may interact with various systems in the body and given the limited in vivo scientific data available on *E. tirucalli*, a significant gap remains in understanding the influence of its latex on critical systems such as the cardiovascular system, particularly in healthy individuals.

Using doses aligned with traditional usage, we investigated the effects of *E. tirucalli* latex on cardiovascular function, focusing specifically on cardiac hemodynamics. Cardiac performance, particularly the heart’s function as a pump, is crucial for maintaining proper organ perfusion and systemic homeostasis. Key physiological parameters such as energy metabolism, blood volume, body temperature, and pH are closely linked to cardiovascular function [16,17].

Arterial pressure is a fundamental indicator of cardiovascular health. In this study, we employed tail-cuff plethysmography—an effective, noninvasive method—to assess blood pressure at baseline and after 15 days of *E. tirucalli* treatment. No significant changes were observed. However, recognizing the limitations of indirect measurement due to variables such as stress and environmental conditions [18], we also performed direct measurements of left ventricular pressure.

Direct hemodynamic assessment revealed that *E. tirucalli* treatment significantly increased both systolic (LVSP) and end-diastolic (LVEDP) left ventricular pressures. These parameters reflect the efficiency of cardiac contraction and the heart’s ability to fill during diastole, respectively [19,20,21,22]. Elevated values, such as those observed here, may suggest increased myocardial workload and ventricular wall stress, potentially indicating early-stage hypertrophy or impaired relaxation; however, no significant differences in heart weight were observed between groups.

Given the short treatment duration and absence of changes in systemic arterial pressure, we interpret the increased intraventricular pressures as indicative of elevated cardiac workload rather than pathological hypertrophy. It is plausible that *E. tirucalli* latex components influence myocardial contractility. In particular, phorbol esters—compounds found in latex—are known to mimic diacylglycerol (DAG) and activate protein kinase C (PKC), a pathway implicated in modulating myocardial contractility [23].

We recently demonstrated that *E. tirucalli*, administered under the same experimental conditions, induces marked alterations in renal hemodynamics [24]. To our knowledge, this is the first study to document the direct in vivo cardiac hemodynamic effects of *E. tirucalli*. Although some reports have indicated that other species within the same genus may reduce blood pressure in healthy animals [25], our findings show that *E. tirucalli*, when delivered at traditional doses, significantly affects ventricular ejection dynamics and myocardial efficiency.

To better characterize these effects, we evaluated contractility and relaxation parameters throughout the cardiac cycle. We observed a significant increase in cardiac cycle duration, suggestive of altered myocardial performance. The maximal rate of pressure development (dP/dT_max_), a well-established marker of contractile function, was significantly increased, indicating enhanced myocardial inotropy [26]. Conversely, the relaxation time constant, Tau, a sensitive and preload-independent index of diastolic function, was reduced [22,27], supporting the hypothesis of altered ventricular compliance or adaptive changes in workload. However, it remains unclear whether these alterations reflect a direct effect on the myocardium or are secondary to systemic and/or renal dysfunction previously described under similar experimental conditions [24]. Therefore, further studies are required to clarify the relative contributions of these mechanisms and to better define the pathways involved.

In addition to functional assessments, we explored possible biochemical mechanisms underlying these hemodynamic alterations. Oxidative stress is a known contributor to cardiovascular dysfunction. Our findings revealed significantly elevated levels of advanced oxidation protein products (AOPPs), indicating increased protein oxidation in cardiac tissue. Given the contractile protein composition of cardiomyocytes and the critical role of membrane proteins in cardiac electrophysiology, protein oxidation may impair myocardial function [28,29,30].

We also detected a significant increase in myeloperoxidase (MPO) activity, a marker of inflammation and oxidative injury [31,32]. The concurrent elevation of AOPP and MPO supports the hypothesis that *Euphorbia tirucalli* latex exerts pro-oxidant and pro-inflammatory effects, as previously demonstrated by our group [24]. Although some Euphorbiaceae species have been reported to reduce MPO activity [33], these differences may be attributed to distinct phytochemical profiles among species. For instance, species such as *Manihot esculenta* are rich in flavonoids and other phenolic compounds, which are commonly associated with antioxidant and anti-inflammatory properties. In contrast, *Euphorbia tirucalli* latex contains predominantly diterpene esters, compounds frequently linked to pro-inflammatory and cytotoxic effects. These compositional differences may underlie the divergent MPO responses observed, reinforcing the concept of species-specific biological activity within the Euphorbiaceae family.

To further substantiate these findings, we quantified hydrogen peroxide (H_2_O_2_), a key reactive oxygen species (ROS), and observed elevated levels in both cardiac cells and circulating leukocytes of treated animals. Interestingly, this finding contrasts with previous reports showing reduced oxidative stress and inflammatory markers following treatment with *Euphorbia bicolor* extracts [29]. Such divergence may reflect differences in experimental conditions, including the specific *Euphorbia* species used, the nature and preparation of the extract, and the biological model and endpoints evaluated.

Importantly, increased ROS production can lead to apoptosis, particularly via oxidative DNA damage and mitochondrial dysfunction [34]. Using Annexin-V staining, we demonstrated a significant rise in early apoptotic cardiomyocytes following *E. tirucalli* treatment. This is consistent with the existing literature reporting that Euphorbiaceae-derived compounds induce apoptosis in tumor cells, possibly via PKC activation [35].

We therefore hypothesize that *E. tirucalli*-induced cardiac effects are mediated by a combined mechanism involving PKC activation and ROS generation, both known to influence apoptotic signaling and myocardial function [23]. However, this hypothesis remains to be experimentally confirmed, as the present study did not include a direct evaluation of PKC activation (e.g., Western blotting using anti-PKC antibodies), which constitutes a limitation. Future studies should address this aspect to further strengthen the mechanistic interpretation of the findings. In addition, efforts should be directed toward the isolation of compounds identified as phorbol esters, as well as their controlled hydrolysis, in order to elucidate their direct contribution to the biological effects observed in the present study.

Collectively, our findings suggest that *Euphorbia tirucalli*, even when administered at doses consistent with traditional use, may alter cardiac hemodynamics, increase oxidative stress, and be associated with early myocardial apoptotic events. These effects may involve bioactive compounds such as phorbol esters and potential PKC-related pathways; however, further mechanistic studies are required to confirm these interactions. Taken together, our results highlight the need for caution in the indiscriminate use of this plant, particularly in individuals with pre-existing cardiovascular conditions.

## 4. Materials and Methods

### 4.1. Plant Material Preparation and Extraction

Latex from *Euphorbia tirucalli* was collected on 11 March 2019, at 9:00 a.m. in Vila Velha, Espírito Santo, Brazil (20.3778279° S, 40.3063419° W). Six drops of latex (13.47 mg/kg) were transferred into microtubes previously containing 500 µL of distilled water. The administered dose was calculated based on previously determined physicochemical properties of the latex, including average volume per drop (20 μL) and density (1.0866 g/mL), allowing conversion from volume to mass. Dose selection was further based on traditional human use of *Euphorbia tirucalli* and converted to an equivalent rat dose using body surface area (BSA) scaling, as previously described by our group [24]. The samples were stored at 4 °C until further use. A voucher specimen was botanically identified and deposited in a recognized herbarium under registration number RFA31675.

A portion of the collected samples was subsequently sent to the Chromatography Laboratory at the Núcleo de Competências em Química do Petróleo (NCQP), Federal University of Espírito Santo (UFES), for chemical characterization of the *E. tirucalli* latex. The samples were analyzed using gas chromatography coupled to mass spectrometry (GC–MS, Shimadzu Corporation, Kyoto, Japan) in order to determine the chemical constituents present in the latex. Additionally, the samples were subjected to high-resolution mass spectrometric analysis under electrospray ionization in negative mode using Fourier transform ion cyclotron resonance mass spectrometry (ESI(–) FT-ICR MS, Bruker Corporation, Billerica, MA, USA). Compounds were assigned based on accurate mass measurements, and their relative abundance was estimated from signal intensity, expressed as percentage values. These values should be interpreted as relative (semi-quantitative) abundances, as absolute quantification would require the use of analytical standards and calibration curves.

### 4.2. Experimental Animals and Study Design

Six-month-old male Wistar rats were obtained from the animal facility of the Health Sciences Center, Federal University of Espírito Santo (UFES), Brazil. The animals were maintained under controlled environmental conditions (22 °C; 12 h light/dark cycle) with free access to standard chow and water. All experimental procedures were conducted in accordance with the guidelines of the National Council for Animal Experimentation Control (CONCEA), and the protocol was approved by the Institutional Ethics Committee on Animal Use (CEUA-UFES, protocol no. 01/2022).

Animals were randomly assigned into two groups (*n* = 8 per group): (1) control, which received 1 mL of distilled water daily by oral gavage; and (2) Aveloz, treated with *Euphorbia tirucalli* latex (13.47 mg/kg diluted in 1 mL of distilled water) administered daily by gavage for 15 consecutive days. At the end of the treatment period, animals were anesthetized via intraperitoneal injection of ketamine (100 mg/kg; Agener, São Paulo, Brazil) and xylazine (10 mg/kg; Bayer, São Paulo, Brazil) for hemodynamic assessments. Subsequently, plasma and cardiac tissue samples were collected for the determination of biochemical markers associated with oxidative stress, inflammation, and apoptosis.

### 4.3. Noninvasive Blood Pressure Assessment

Systolic blood pressure (SBP) was measured in conscious, restrained rats using a noninvasive tail-cuff plethysmography system (IITC Life Science Inc., Woodland Hills, CA, USA). To minimize stress, animals were habituated to the procedure one day prior to data collection. During measurements, rats were placed in restrainers on a heated platform (35 °C) to induce tail vasodilation. A photoelectric sensor-coupled tail cuff was positioned at the tail base to detect pulse waves, which were amplified and converted to digital signals. After a 5 min acclimation, ten inflation–deflation cycles were recorded, and the mean SBP was calculated from the final five cycles, as described by Melo Junior et al. [36].

### 4.4. Invasive Hemodynamic Evaluation

Immediately after the SBP measurements, rats were anesthetized, and the right common carotid artery was surgically exposed and cannulated with a saline-filled polyethylene catheter (PE-50). The catheter was connected to a pressure transducer (Cobe Laboratories, Lakewood, CO, USA) and a data acquisition system (MP100, Biopac Systems, Goleta, CA, USA) to monitor blood pressure (BP) and heart rate (HR).

After stabilization, the catheter was advanced into the left ventricle (LV) to assess cardiac function parameters, including left ventricular systolic pressure (LVSP), left ventricular end-diastolic pressure (LVEDP), maximum and minimum rates of pressure change (dP/dT_max_ and dP/dT_min_), cardiac cycle duration, and the relaxation time constant (Tau). Data were recorded and analyzed using LabChart software v 7.3.8 (ADInstruments, Castle Hill, Australia). Following LV catheterization, the catheter was retracted to reassess aortic pressure and confirm aortic valve integrity [36].

### 4.5. Plasma Oxidative Stress and Inflammatory Activity

After hemodynamic assessments, animals were euthanized with an overdose of thiopental (100 mg/kg, i.p.; Cristália, São Paulo, Brazil). Blood samples were collected by cardiac puncture and subsequently centrifuged for plasma separation, which was used for oxidative and inflammatory analyses. Advanced oxidation protein products (AOPPs) were quantified spectrophotometrically according to the method by Witko-Sarsat et al. [37], using chloramine-T as the standard.

Plasma samples were diluted 1:200 in phosphate buffer (20 mM, pH 7.4), and 40 µL of each diluted sample was mixed with phosphate-buffered saline (PBS), potassium iodide (1.16 M), and acetic acid in a 96-well microplate. After 6 min of agitation, absorbance was measured at 340 nm using a microplate reader (BioTek^®^ Synergy H1, Winooski, VT, USA). AOPP concentrations were expressed as the ratio of oxidized to total proteins, with total protein content determined using the Bradford method at 595 nm [38].

Myeloperoxidase (MPO) activity, used as an inflammatory marker, was determined according to the method described by Bradley et al. [39]. Briefly, 12 µL of plasma was incubated with 236 µL of o-dianisidine reagent (0.167 mg/mL, Sigma-Aldrich, St. Louis, MO, USA) and hydrogen peroxide (0.0005%) in PBS (50 mM, pH 6.0). Absorbance was measured at 460 nm over 15 min, and MPO activity was expressed as units of enzymatic activity.

### 4.6. Assessment of Reactive Oxygen Species (ROS) and Apoptosis in Circulating Leukocytes and Cardiac Cells

All analyses were performed using primary cells freshly isolated from rat blood and cardiac tissue; no established or immortalized cell lines were used in this study. Prior to perfusion, blood was collected via cardiac puncture, and erythrocytes were lysed using a saline-based lysis solution, followed by storage of circulating leukocytes in 95% fetal bovine serum (FBS). The heart was then excised and weighed, and the left ventricle (LV) was sectioned into ~150 mg fragments, which were enzymatically dissociated using collagenase type I (Gibco, Waltham, MA, USA) as described by Louch et al. [40], with minor modifications. Tissue digestion was performed at 37 °C for 60 min in a shaker incubator (1200 rpm) protected from light. The resulting cell suspension was filtered through a 70 µm nylon mesh and washed three times with FBS.

Reactive oxygen species (ROS) production was determined in circulating leukocytes and cardiac cells using 2′,7′-dichlorofluorescein diacetate (DCF-DA, Sigma-Aldrich, St. Louis, MO, USA), whereas apoptosis in cardiac cells was assessed using annexin V-FITC (Sigma-Aldrich, St. Louis, MO, USA) and propidium iodide (PI), following Tonini et al. [41]. For ROS analysis, 10^6^ cells were incubated with 20 µM DCF-DA for 30 min at 37 °C in the dark, washed, and resuspended in PBS. Fluorescence was measured using a FACSCanto II flow cytometer (Becton Dickinson, BD, San Diego, CA, USA) with excitation at 488 nm and emission collected through a 530/30 nm bandpass filter. Results are expressed as median fluorescence intensity (MFI). Cells treated with 50 mM H_2_O_2_ served as positive controls, and those treated with ethanol were negative controls.

For apoptosis detection, 10^6^ cardiac cells were centrifuged, resuspended in binding buffer (10 mM HEPES/NaOH, 140 mM NaCl, 2.5 mM CaCl_2_), and stained with 5 µL annexin V-FITC and 5 µL propidium iodide (PI) for 15 min at room temperature in the dark. After the addition of 400 µL of binding buffer, samples were analyzed within 1 h using a flow cytometer. A minimum of 10,000 events was acquired per sample. Cells positive for Annexin V and negative for PI (Annexin V^+^/PI^−^) were considered early apoptotic, and data are expressed as the percentage of total cells.

In all experimental protocols, a total of 10,000 events were acquired. Instrument settings, including nozzle size and flow rate, were optimized according to the size and morphological characteristics of the different cell populations analyzed in the ROS and apoptosis assays. The data were analyzed using FlowJo X 10.0.7r2 software (Becton Dickinson, BD, San Diego, CA, USA).

### 4.7. Data Analysis

All data are presented as the mean ± SEM for each group (*n* = 8–10 per group). The normality of data distribution was assessed using the Kolmogorov‒Smirnov test. Since all samples exhibited a Gaussian distribution, an unpaired Student’s *t*-test was used for statistical analysis after *E. tirucalli* administration. *p*-values less than 0.05 (*p* < 0.05) were considered statistically significant. Statistical analyses were performed using GraphPad Prism version 9.0 (GraphPad, Inc., San Diego, CA, USA).

## 5. Conclusions

In summary, the present study provides an initial in vivo characterization of the cardiovascular effects of *Euphorbia tirucalli* latex administered at doses consistent with traditional use. Our findings indicate that exposure to this plant may be associated with alterations in cardiac hemodynamics, increased oxidative stress, and early apoptotic events in cardiomyocytes. While these effects may involve bioactive compounds such as phorbol esters and pathways related to PKC activation, the underlying mechanisms remain to be fully elucidated. Therefore, further studies are required to confirm these pathways and to better define the molecular mechanisms involved. Overall, these findings contribute to a more comprehensive understanding of the biological effects of *E. tirucalli* and reinforce the need for cautious use of this plant, particularly in individuals with potential cardiovascular vulnerability.

## Figures and Tables

**Table 1:** Diterpene esters identified in the crude extract of *E. tirucalli* latex by ESI(–) FT-ICR MS.

[M-H]^−^	Molecular Formula	Intensity (%)	Error (ppm)	DBE	Proposed Compound
495.27586	C_30_H_39_O_6_	1.6	−1.31	11.0	Ingenol 3-(2,4,6-decatrienoate)
497.29151	C_30_H_41_O_6_	0.58	−1.31	10.0	Ingenol 3-(2,4-decadienoate)
511.27074	C_30_H_39_O_7_	33.94	−1.2	11.0	12-octa-2,4-dienoate, 13-acetate-4-deoxyphorbol
531.25258	C_30_H_40_ClO_6_	9.71	−1.3	11.0	Chloride adduct of ingenol 3-(2,4,6-decadienoate)
547.24743	C_30_H_40_ClO_7_	100	−1.15	11.0	Chloride adduct of 12-octa-2,4-dienoate, 13-acetate-4-deoxyphorbol
557.26832	C_32_H_42_ClO_6_	3.41	−1.4	12.0	Chloride adduct of ingenol 3-(2,4,6,8-dodecatetraenoate)
559.28411	C_32_H_44_ClO_6_	2.43	−1.64	10.0	Chloride adduct of ingenol 3-(2,4,6-dodecadienoate)
583.28411	C_34_H_44_ClO_6_	0.96	−1.57	13.0	Chloride adduct of ingenol 3-(2,4,6,8,10-tetradecapentaenoate)

## Data Availability

The datasets generated and/or analyzed during the current study are not publicly available due to institutional and ethical regulations regarding experimental animal research (approved by the Institutional Animal Care and Use Committee, CEUA-UFES Register number 01/2022. However, the data are available from the corresponding author upon reasonable request.
